# Tumor Biomarkers for the Prediction of Distant Metastasis in Head and Neck Squamous Cell Carcinoma

**DOI:** 10.3390/cancers12040922

**Published:** 2020-04-09

**Authors:** Salvatore Alfieri, Andrea Carenzo, Francesca Platini, Mara S. Serafini, Federica Perrone, Donata Galbiati, Andrea P. Sponghini, Roberta Depenni, Andrea Vingiani, Pasquale Quattrone, Edoardo Marchesi, Maria F. Iannó, Arianna Micali, Elisa Mancinelli, Ester Orlandi, Sara Marceglia, Laura D. Locati, Lisa Licitra, Paolo Bossi, Loris De Cecco

**Affiliations:** 1 Head and Neck Cancer Medical Oncology 3 Department, Fondazione IRCCS Istituto Nazionale dei Tumori (INT), 20133 Milan, Italy; salvatore.alfieri@istitutotumori.mi.it (S.A.); francesca.platini@istitutotumori.mi.it (F.P.); donata.galbiati@istitutotumori.mi.it (D.G.); laura.locati@istitutotumori.mi.it (L.D.L.); paolo.bossi@istitutotumori.mi.it (P.B.); 2 Integrated Biology Platform, Department of Applied Research and Technology Development, Fondazione IRCCS Istituto Nazionale dei Tumori, 20133 Milan, Italy; andrea.carenzo@istitutotumori.mi.it (A.C.); Mara.Serafini@istitutotumori.mi.it (M.S.S.); edoardo.marchesi@istitutotumori.mi.it (E.M.); Maria.Ianno@istitutotumori.mi.it (M.F.I.); Arianna.Micali@istitutotumori.mi.it (A.M.); elisa.mancinelli@istitutotumori.mi.it (E.M.); 3 Pathology Department, Fondazione IRCCS Istituto Nazionale dei Tumori, 20133 Milan, Italy; Federica.Perrone@istitutotumori.mi.it (F.P.); Andrea.Vingiani@istitutotumori.mi.it (A.V.); Pasquale.Quattrone@istitutotumori.mi.it (P.Q.); 4 Oncology Department, University of Eastern Piedmont, Maggiore della Carità Hospital, 28100 Novara, Italy; asponghini@libero.it; 5 Department of Oncology and Haematology, University Hospital of Modena and Reggio Emilia, 41124 Modena, Italy; depenni.roberta@policlinico.mo.it; 6 Radiation Oncology Department, Fondazione IRCCS Istituto Nazionale dei Tumori, 20133 Milan, Italy; ester.orlandi@istitutotumori.mi.it; 7 Department of Engineering and Architecture, University of Trieste, 34127 Trieste, Italy; smarceglia@units.it; 8 Department of Hematology and Oncology, University of Milan, 20122 Milan, Italy

**Keywords:** biomarkers, distant metastases, gene expression profiling, head and neck squamous cell carcinoma

## Abstract

Distant metastases (DM) in head and neck squamous cell carcinoma (HNSCC) remain a challenge as treatment options are limited. To identify biomarkers predictive of DM in primary tumors (PT), gene expression profiling was performed in PT from patients who did, or did not develop DM (T-with and T-without, *n* = 25 and 24, respectively), and in matched DM. A total of 185 and 42 differentially expressed genes were identified in the T-with vs. T-without and the T-with vs. DM comparisons, respectively. The intersection between these two comparisons identified COX7A1 and TBX5 as common genes. In three independent datasets, both genes were able to significantly distinguish patients according to their DM-free survival. By functional biological analyses, the T-without group showed enrichment in immune-response pathways, whereas the T-with group showed an enrichment in B-plasma cells and Tregs. Increased enrichment of proliferation-related pathways was observed in the T-with group compared with that in the DM group. Further comparisons with/without DM are needed to confirm these data in order to improve clinical management of HNSCC.

## 1. Introduction

Head and neck squamous cell carcinoma (HNSCC) is a heterogeneous group of malignancies arising from different sites, including the oral cavity, hypopharynx, oropharynx, and larynx [1]. The anatomical location and the tumor-node-metastasis (TNM) staging score system are used as reference guides for prognosis and the selection of treatments. While the advent of multimodality therapies has significantly improved the outcomes of locally advanced HNSCC patients, the appearance of distant metastases (DM) remains a challenge for prognosis [2]. Currently, about 10% to 20% of all HNSCC patients develop DM [3], and it is rarely observed at first diagnosis; in the majority of metastatic patients, the development of a DM occurs after curative first line treatment for loco-regional disease. DMs are still detected during follow-up despite the refinements that have occurred over the last decade in new screening modalities at initial diagnosis: 50% of DMs are clinically detected within 9 months of treatment and 80% within 2 years [4]. The most frequent DM sites in HNSCC patients are the lungs, followed by the less common liver and bone. It is essential to keep in mind that the risk of DM is correlated with initial staging. Moreover, DMs are often missed considering that from 24% to 42% of them are found at autopsy in HNSCC patients staged as being clinically node-negative [5]. Till now, recognized clinical and biological predictors of increased risk of DM are hypopharyngeal sub-site, older age, smoking status, N3 and T4 disease, and a negative p16 status. However, this risk stratification model is limited and its value may be improved by adding the biologic characteristics of the primary tumor (PT) to better elucidate the propensity of metastatization of HNSCC [6].

Over the last two decades, a limited number of studies have identified predictive transcriptomic signatures for the development of metastasis. In these studies, PTs were collected from patients with a follow-up of at least 36 months in order to compare: (i) matched samples of non-metastasizing and metastasizing cases as in Cromer et al. [7]; (ii) samples from patients who developed DM and non-metastatic control cases as in Braakhuis et al. [8]; (iii) samples of both “high-risk” DM patients and non-metastatic patients as in Giri et al. [9]. Although each study reported unique expression patterns involved in the acquisition of metastatic potential, small sample sizes and the lack of statistically significant results and of independent validation have prevented the validation of any of these predictive biomarkers. Later on, Rickman et al. [10] reported a study in a larger cohort of PTs, collected from patients treated with surgery who developed (or not) metastases as the first recurrent event. Rickman et al. [10], as first, proposed a predictive signature for patients who developed DM within 36 months of follow-up retaining an independent value when challenged in multivariate analysis against known clinical factors. The risk of DM development has also been evaluated in PTs at the single gene biomarker level by immunohistochemistry (IHC). Researchers have identified a number of biomarkers that mirror molecular alterations in specific pathways but, at present, none of these has been used in the clinical management of patients. For example, (i) TWIST expression was found to be correlated with low tumor differentiation and DM occurrence [11]; (ii) pAkt and survivin have been found to be positively associated with DM [12]; and (iii) a down-modulation of E-cadherin expression has also been correlated with the risk of DM [13]. 

In order to identify specific biomarker(s) that are expressed in PTs and be able to predict the DM development, we exploited a bioinformatics approach that estimates the area under the receiver operating characteristic (ROC) curve (AUC), a common measure of discrimination, and the overlapping coefficient (OVL) between two densities [14]. ROC has been extensively used in translational research studies to assess the effectiveness of continuous biomarkers in distinguishing between two disease conditions, enabling the selection of an optimal threshold value. However, as a result of the biological properties of the tumor cells, the biomarker values could follow multi-modal data distributions that traditional diagnostic indices (i.e., AUC) fail to capture. In this way, to gain a more comprehensive evaluation of the biomarker performances, we should implement methods able to detect differences in the shape and scale of data distributions, other than their mean/median values; therefore, the OVL can be used as an index to assess the overlap area between data densities. The method introduced by Silva-Fortes et al. [14] allows a summary of the AUC and OVL in gene expression data for identifying specific genes as valuable biomarkers.

With the final goal of identifying biomarkers that could be used to predict patients who will develop DM and to explore the biology behind metastasis, we selected HNSCC patients applying a comparison of matched PTs in patients who did (“T-with”) and did not (“T-without”) develop DM without an a priori selection/exclusion of human papilloma virus (HPV) status, DM site, and the presence of loco-regional disease at diagnosis. Additionally, we collected tissue samples from the DM in the T-with patients. Despite the efforts made in several years of translational research, the available data still remain as potential findings; our study will provide a framework to test the available biomarkers and to develop feasible models that hopefully will be able to improve clinical management in the near future.

## 2. Results

### 2.1. Study Population: Recruitment and Clinical Characteristics

**Figure 1:**
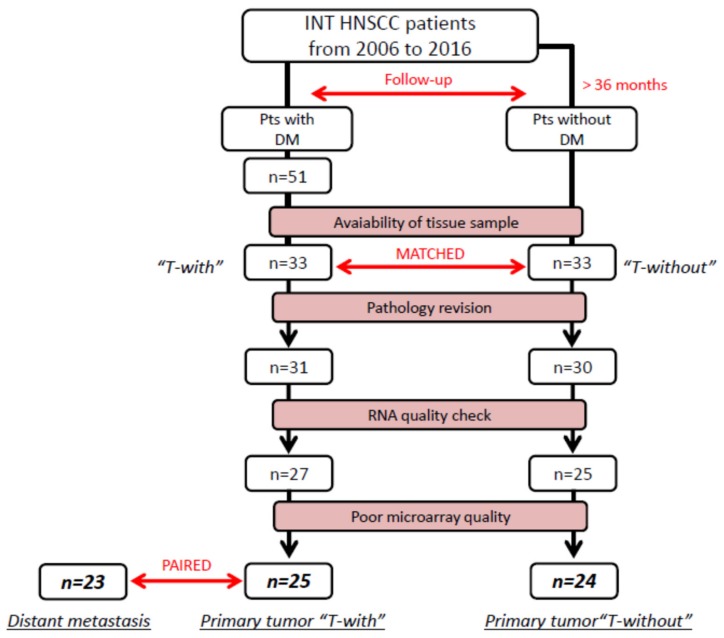
Study population recruitment. Sample selection criteria resulting in 25 T-with and 24 T-without tumors available for gene expression analysis.

Among the HNSCC patients treated with curative intent at the Fondazione IRCCS Istituto Nazionale dei Tumori (INT) in Milan from 2006 to 2016, 51 patients had developed DM with or without loco-regional recurrence (Figure 1). Primary tissue samples of T-with patients were not available in 18 cases, and were excluded. According to the clinical characteristics of the T-with group, a group of 33 patients, who did not develop metastasis (follow-up >36 months) and for whom primary tissues were available, were selected as the T-without group for comparison. Among 33 primary tissue samples for each of the T-with and T-without groups, further samples were excluded as follows: after pathology revision (<70% of tumor cells), 2 T-with and 3 T-without cases, and after RNA extraction (poor RNA quality/quantity), 4 T-with and 5 T-without samples. Finally, after a quality check of the microarray data, the final cohort included 25 T-with and 24 T-without cases. Additionally, for 23 of the 25 selected T-with cases, we were able to collect matched formalin-fixed paraffin-embedded (FFPE) samples of the DM (Figure 1). The demographic and clinical characteristics are shown in Table 1. The T-with and T-without groups were well balanced with respect to age, sex, smoking history, and primary disease subsite. Based on the HPV status in the oropharynx cancer patients, 6 out of 7 and 5 out of 8 were HPV positive in the T-with and T-without group respectively (*p*-value = 0.2). Patients were also balanced in accordance with their TNM, VIII edition, classification: 7 patients were classified as stage I–II, and 18 patients as stage III–IV in the T-with group, whereas 5 patients were classified as stage I–II, and 19 patients as stage III–IV in the T-without group. Of note, 16/25 T-with patients and 14/24 T-without patients had lymph-node involvement at the time of diagnosis (Supplementary Figure S1). At diagnosis, synchronous metastasis was detected in 1 patient (HPV positive oropharynx, stage IV). At follow-up, 52% of T-with patients had lung metastases (13/25), 12% had liver metastases (3/25), and 36% had metastases in other sites (9/25: 4 distant lymph-nodes; 2 skin and soft tissues; 1 kidney; 1 adrenal gland; 1 pleura). Additionally, 12/25 T-with patients had loco-regional recurrence before or concomitantly with DM development (8 in the lymph-nodes and 4 in the PT) (Supplementary Figure S1). The median time to DM onset was 16 months (range 3–59), and the median follow-up time for the T-without group was 67 months (range 48–106) (Table 1). Radiotherapy of the head and neck area was performed in 18/25 (72%) of the T-with group and 19/24 (79%) of the T-without group. Systemic treatment (chemotherapy or cetuximab) was performed concomitantly to radiation with curative intent in 15/25 (60%) of the T-with patients, and in 12/24 (50%) of the T-without patients. Surgery was part of the curative treatment in 17/25 (68%) of T-with and in 15/24 (62%) of T-without.

### 2.2. Assessing the Performance of Existing Biomarkers

Our cohort was profiled using a whole transcriptome microarray platform. Initially, we used our data to independently test the potential available biomarkers whose association with the DM development in HNSCC had been reported in previous studies. Rickman et al. [10] reported a gene expression signature that was able to stratify patients based on distant metastasis-free survival (DMFS). We tested the ability of 17 of their 19 genes to stratify the T-with and T-without cases, reaching an AUC = 0.63 (95% CI: 0.469–0.791) (Supplementary Figure S2). A number of other reports have identified biomarkers useful for predicting the metastatic potential of HNSCC. After a survey of literature, we created a list of 19 genes from 16 studies that were tested in our case material; three of these genes reached an AUC > 0.6 (ANXA2, BDNF, and TGFB1 with AUCs = 0.605, 0.722, and 0.717, respectively) (Supplementary Table S1).

### 2.3. Identification of Differentially Expressed Genes in the T-with, T-without, and Paired Distant Metastasis Samples by AUC and OVL

**Figure 2:**
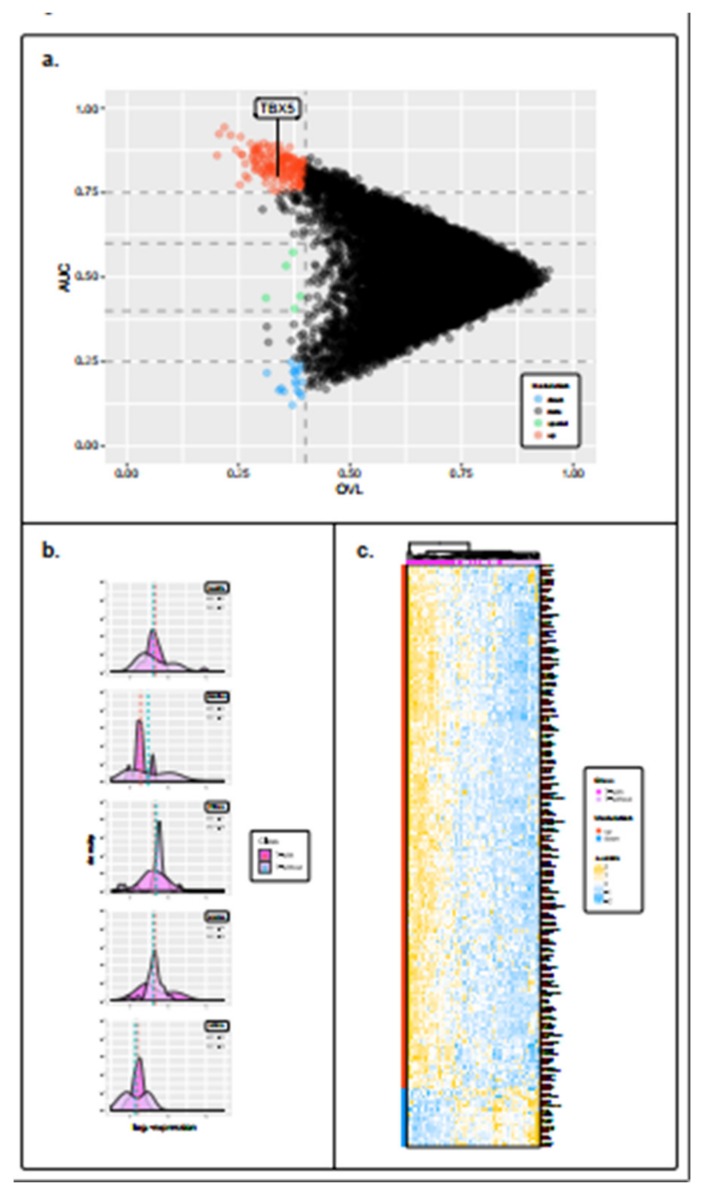
Identification of significantly differentially expressed genes between the T-with and T-without tumors. (**a**) Arrow plot showing genes in the OVL–AUC plane, whose dimensions represent the overlapping coefficient and the area under the ROC curve, respectively. We considered as genes of interest those with an OVL < 0.4. Specifically, these were genes with an AUC > 0.75 (up-regulated in the T-with group, red dots) or genes having an AUC < 0.25 (down-regulated in the T-with group, blue dots). Genes having 0.4 < AUC < 0.6 are referred to as special (green dots), since they had the same mean expression levels between the T-with and T-without groups, but also had very different data distributions. (**b**) Density plots showing the distributions of the five special genes (NUDT6, COX7A1, TTC19, SNRPA1, and UFD1L). (**c**) Heatmap clustered by Euclidean distance and the complete linkage method representing gene expression levels of the up- and down-regulated genes in the T-with and T-without groups. On the left of the figure the red and blue bars specify whether the genes are up- or down-regulated respectively, while on the top, the magenta and light pink squares indicate whether the samples belong to the T-with or T-without groups respectively. Gene expression levels colors range from blue (low expression), to white (mild expression), to yellow (high expression).

The AUC and OVL between the T-with and T-without groups were assessed, in order to evaluate whether clinically relevant biomarkers could be disclosed or if we could uncover gene signatures that could be used to predict DM. The results were visualized through an arrow plot that identified three categories of genes (i.e., up- or down-regulated and special genes) (Figure 2a). Following the arrow plot procedure, we were able to identify a total of 185 differentially expressed genes in the T-with group compared to the T-without group: 162 up-regulated, 18 down-regulated, and 5 special genes (Figure 2b and Supplementary Table S2). The heatmap in Figure 2c depicts the expression levels of these differentially expressed genes in the T-with and T-without groups.

**Figure 3:**
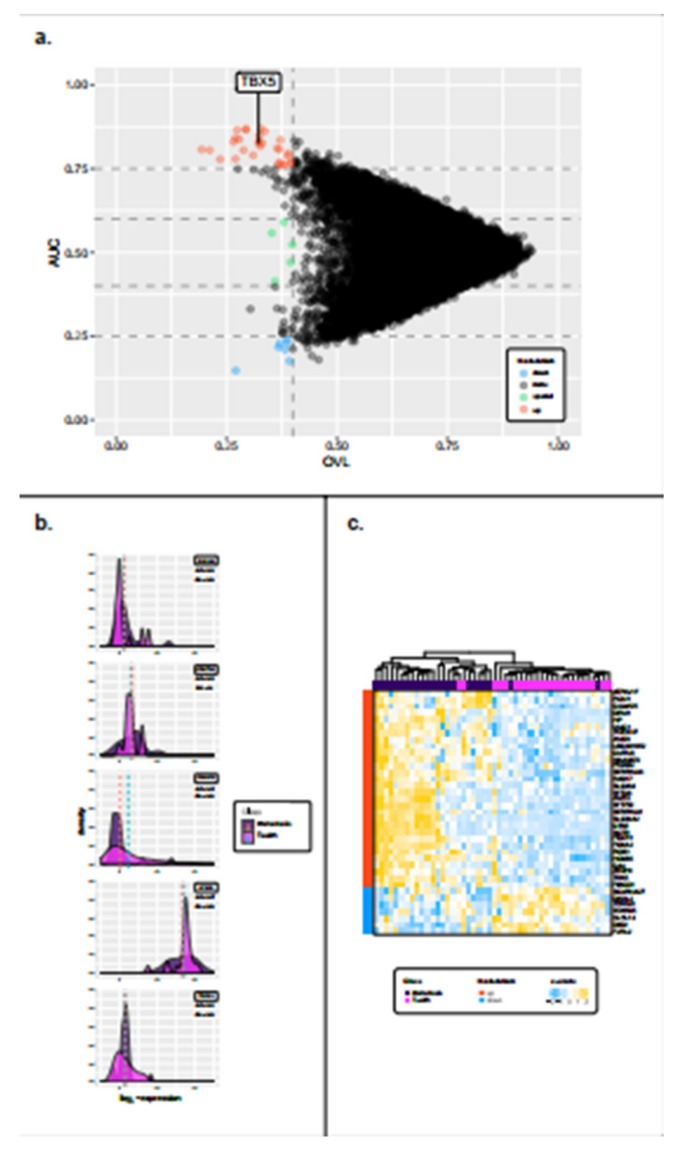
Identification of significantly regulated genes in T-with tumors compared to paired DM tumors. (**a**) Arrow plot showing genes in the OVL–AUC plane. Genes with an OVL value less than 0.4 are considered to be of interest. Those having an AUC > 0.75 are considered to be up-regulated in the metastasis group (red dots), whereas those having an AUC < 0.25 (below the lower horizontal dashed line) are considered to be down-regulated (blue dots). Genes having 0.4 < AUC < 0.6 (those lying between the second and third horizontal dashed lines) are the special genes (green dots). (**b**) Density plots showing the distribution of the five special genes (ZNF491, COX7A1, CRABP1, EP300, TRPC4). (**c**) Heatmap clustered by Euclidean distance and the complete linkage method representing gene expression levels for the up- and down-regulated genes in the primary and metastasis groups. On the left of the figure, the red and blue bars specify whether the genes are up- or down-regulated respectively, while on the top, the purple and magenta squares indicate if samples belong to the metastasis or primary tumors groups, respectively. Gene expression levels colors range from blue (low expression), to white (mild expression), to yellow (high expression).

We also explored the molecular differences between 23 T-with samples and their paired DMs. In comparing T-with and matched DMs we applied the arrow plot procedure used in the previous analysis, maintaining the same thresholds, and we identified 30 up-regulated, 7 down-regulated (Figure 3a), and 5 special genes (Figure 3b). The heatmap in Figure 3c depicts the expression levels of these differentially expressed genes in the T-with and DM groups (Supplementary Table S2).

### 2.4. Evaluation of 2 Differentially Expressed Genes in Independent Datasets

**Figure 4:**
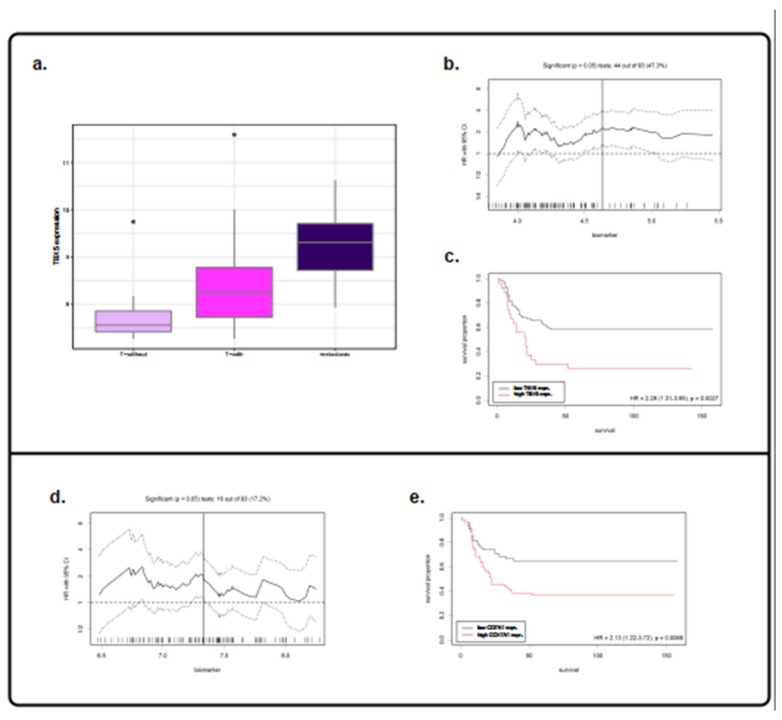
TBX5 expression levels and TBX5 and COX7A1 evaluation in Cromer–Rickman combined dataset. (**a**) The boxplots depict the expression of TBX5 in the T-without, T-with, and distant metastasis (DM) tumors. (**b**) Optimal cutoff values using Cutoff Finder for TBX5. The plot shows the hazard ratio (HR) for DM-free survival (DMFS) at various cutoff points of TBX5 expression values. The vertical line defines the optimal cutoff value, as the point with the most significant HR. The solid and broken lines indicate the HR and the 95% confidence intervals for each HR, respectively. (**c**) Kaplan–Meier curves for TBX5 expression divided based on the best cutoff. The black and red lines indicate the group with low and high values, respectively. (**d**) Optimal cutoff values via Cutoff Finder for COX7A1. The plot shows the HR. (**e**) Kaplan–Meier curves of COX7A1 expression divided based on the best cutoff.

When we compared the previously identified differentially expressed genes in the T-with vs. T-without analysis and those in the T-with vs. DM analysis, we found a single common gene, namely TBX5, which was characterized by low expression in the T-without group, intermediate expression in the T-with group, and high expression in the DM group (Figure 4a). When we examined the special gene class, COX7A1 was identified as the unique common special gene between these two comparisons (T-with vs. T-without; T-with vs. DM). Thus, we deeply investigated the possible association between TBX5 and COX7A1 expression and DMFS. After a survey of publicly available data, we identified two studies (E-TABM-302 and GSE2379), both reporting HNSCC cohorts, for a total of 112 cases with DMFS data as the clinical endpoint. The aggregated dataset, whose main clinical features are summarized in Supplementary Table S3, was used as an independent evaluation set. By applying the Cutoff Finder tool [15] on the merged dataset, a range of cutoff points were imposed on TBX5 expression considering DMFS as endpoint (Figure 4b). When we considered the optimal threshold based on TBX5 expression levels, the cohort could be divided into high TBX5 expression (*n* = 27, 24.1%) and low expression groups (*n* = 85, 75.9%). A Kaplan–Meier survival analysis confirmed that high TBX5 expression was associated with a short DMFS reaching HR = 2.28 (95% CI: 1.31–3.95), *p*-value = 0.0027 (Figure 4c). In the case of COX7A1, the best splitting threshold using the Cutoff Finder tool identified two clusters of patients having high (*n* = 57) or low (*n* = 55) expression levels of COX7A1, with DMFS as the endpoint (Figure 4d). A Kaplan-Meier survival applied on the optimal cutoff yielded HR = 2.13 (95% CI: 1.33–2.55), *p*-value = 0.0066 (Figure 4e). Moreover, the clinically re-annotated HNSCC data of The Cancer Genome Atlas (TCGA) program were investigated for DM cases with suitable clinical characteristics to test TBX5 and COX7A1 expression in association with DMFS as endpoint. The TCGA dataset gave us the opportunity to ascertain on 128 cases to what degree TBX5 and COX7A1 expression is related to DM (and not to loco-regional involvement). We applied the same workflow used for Rickman and Cromer datasets. Applying Cutoff Finder on TBX5 expression, TCGA could be divided into high expression (*n* = 27, 21.1%) and low expression groups (*n* = 101, 78.9%) and Kaplan–Meier survival analysis confirmed that high TBX5 expression was associated with a shorter DMFS reaching HR = 2.35 (95% CI: 1.31–4.23), *p* = 0.0033. Regarding COX7A1, TCGA was divided into 99 cases (77.3%) with high expression while the remaining 29 (22.7%) had low expression; Kaplan–Meier analysis showed that high COX7A1 expression was associated to worst outcome, HR = 2.68 (95% CI: 1.13–6.36), *p*-value = 0.02 (Supplementary Figure S3). 

In order to more closely understand the role of TBX5 in predicting DM events, we extended our analysis to other tumor types; in particular, we focused on breast cancer, since in these patients DM is the leading cause of death [16]. We used the DMFS information from the KM Plotter [17] database to perform a survival analysis for TBX5, reaching a HR = 1.84 (95% CI: 1.33–2.55), with a *p*-value = 0.00018 (Supplementary Figure S4).

### 2.5. Functional Biological Analysis

**Figure 5:**
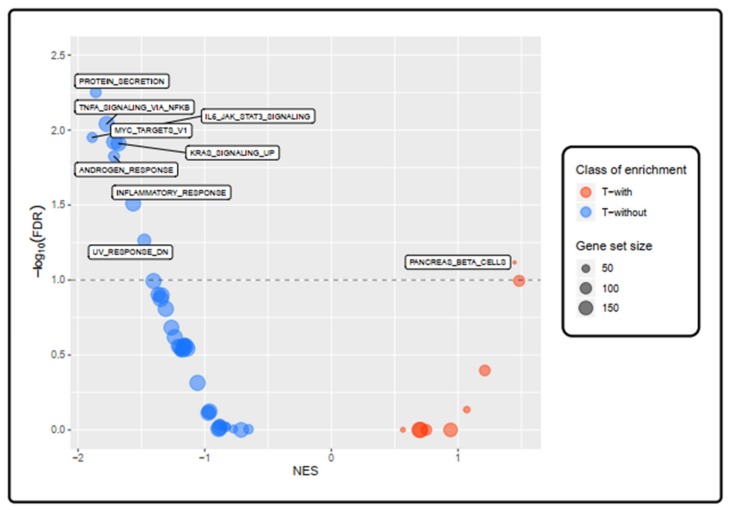
Enriched pathways in T-with tumors compared with T-without tumors: Gene Set Enrichment Analysis (GSEA) Hallmark analysis. Bubble plot showing the enrichment of the Hallmark gene sets between the T-with and T-without groups. Every bubble represents a gene set; the size of the displayed circles is proportional to the number of genes assigned to each term. Red bubbles are gene sets which are more enriched in the T-with group (normalized enrichment score, NES > 0), whereas blue bubbles represent those enriched in the T-without samples (NES < 0). We considered as significantly enriched those gene sets having a False Discovery Rate (FDR) less than 0.1 (10%) and labeled those having a |NES| > 1.4. The higher the y-value of the gene set in the bubble plot (i.e., the smaller its FDR value) and the more extreme its x-value (i.e., NES values), the more the Hallmark gene set is significantly enriched.

To gain further insight into the biological functional characterization of pathways related to the T-with vs. T-without comparison, we performed a Gene Set Enrichment Analysis (GSEA) [18,19]. Imposing a false discovery rate (FDR) < 0.1, and a normalized enrichment score (NES) > 1.4, the T-without group displayed an enrichment in 8 Hallmark gene sets: “IL6 JAK STAT3 SIGNALING”, “PROTEIN SECRETION”, “TNFA SIGNALING VIA NFKB”, “ANDROGEN RESPONSE”, “MYC TARGETS V1”, “KRAS SIGNALING UP”, “INFLAMMATORY RESPONSE”, “UV RESPONSE DN”. Of note, three out of eight enriched Hallmark gene sets (“IL6 JAK STAT3 SIGNALING”, “TNFA SIGNALING VIA NFKB”, “INFLAMMATORY RESPONSE”) correlated with an immune response. In comparison, the T-with group showed a significant enrichment in one Hallmark gene set, namely “PANCREAS BETA CELLS” (Figure 5, Supplementary Table S4).

**Figure 6:**
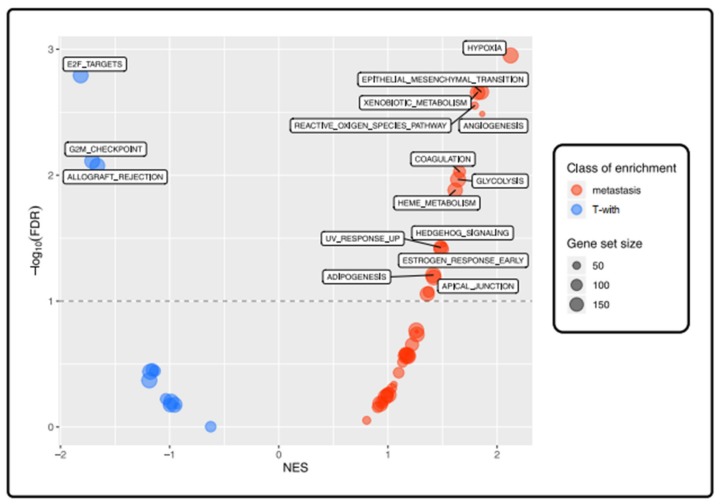
Differently enriched pathways in T-with compared to paired DM samples: GSEA Hallmark analysis. Bubble plot showing the enrichment Hallmark gene sets between the T-with and DM groups. Every bubble represents a gene set; the bigger the size of the bubble, the higher the number of genes in the gene set. Red bubbles are gene sets which are more enriched in the metastasis group (NES > 0), whereas blue bubbles represent those enriched in the primary tumor (PT) samples (NES < 0). We considered as significantly enriched those gene sets having an FDR less than 0.1 (10%) and labeled those having a NES > 1.4.

As with the main comparison, and keeping the same parameters and thresholds, we performed a pre-ranked GSEA between the T-with group and their matched DM specimens. Three Hallmark gene sets, two of them being related to proliferation (“E2F TARGETS”, “G2M CHECKPOINT”), were enriched in the T-with group. A higher number of Hallmark gene sets (N = 13) were found to be enriched in the DM group (Figure 6); most of these gene sets enriched in the DM group were related to a more aggressive/invasive phenotype, such as “HYPOXIA”, “ANGIOGENESIS”, “EPITHELIAL MESENCHYMAL TRANSITION” (EMT), “REACTIVE OXYGEN SPECIES PATHWAY” (ROS), “GLYCOLYSIS” (Supplementary Table S5).

### 2.6. Immune Landscape: CIBERSORT Analysis and PD-L1 Evaluation 

**Figure 7:**
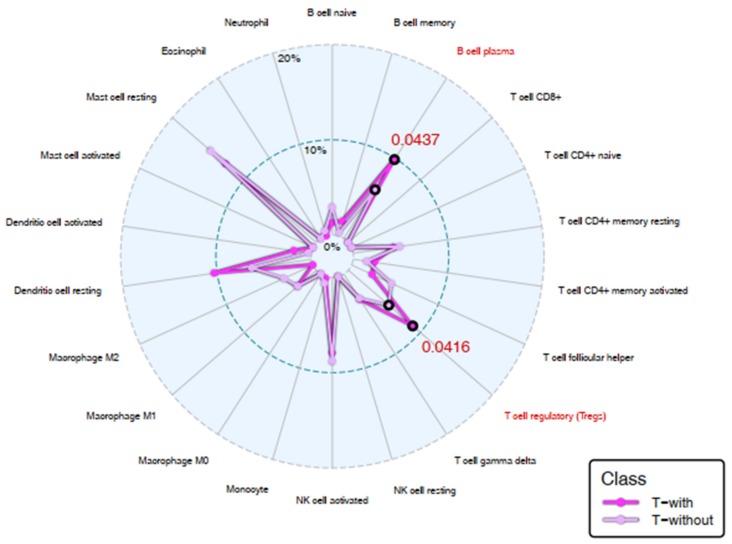
Immune landscape: CIBERSORT analysis. Radar plot showing results obtained after the CIBERSORT procedure. Each portion of the graph represents one of the 22 human cell phenotypes considered in the method, while dots display the mean proportion of each cell population in the T-with (magenta line) and T-without groups (pink line). B cell plasma and T cell regulatory (Tregs) populations (highlighted in red) showed a weak statistically significant difference (*p*-values in red) between the two groups, by performing a Wilcoxon rank sum test with a level of significance of α = 0.05.

We used the CIBERSORT computational tool [20] to estimate the proportion of 22 immune cell populations in each sample. The results of the comparison between the T-with and T-without groups obtained by plotting the mean proportions as a radar plot are shown in Figure 7. We found that there was a significant increase in the plasma B cell and T-regulatory cell (Treg) populations in the T-with group compared with that in the T-without group (*p*-value = 0.0437 and 0.0416, respectively).

Among our case samples, 20 T-with, 21 T-without, and 19 DM samples were available for PD-L1 evaluation by IHC. By comparing PD-L1 expression (as tumor proportion score, TPS) among the two groups (Supplementary Table S6), the T-without samples showed significantly higher levels of expression of PD-L1 in tumor cells than the T-with samples (positive TPS: 45% and 15%, respectively, *p* = 0.048), while no statistically significant difference was found with respect to the inflammatory cells (IC) score and the combined proportion score (CPS). In the matched PT and DM samples (from the T-with group), no significant associations were found for PD-L1 expression (Supplementary Table S6).

With respect to the tumor-infiltrating lymphocytes (TILs), we found a higher, although non-significant, prevalence in the T-without than in the T-with samples (mean: 30.61%, 95% CI: 9.08–52.13%; mean: 23.83%, 95% CI: 2.19–45.47%, respectively, *p* = 0.28), potentially mirroring the PD-L1 status within the two groups. No significant change in TIL levels was found between the T-with and DM groups (Supplementary Table S6).

## 3. Discussion

The appearance of DM in HNSCC remains a challenge for several reasons: the treatment options of DM patients are still limited to chemotherapy, target therapy with epidermal growth factor receptor (EGFR) inhibitors, and immunotherapy [21], life expectancy is dramatically decreased, and quality of life is poor. In this scenario, the availability of a reliable biomarker to predict the risk of DM may help in driving further research and in potentially developing further treatment options. 

In our study, we used a selection procedure for the T-with and T-without groups such that the patients’ characteristics in each group were well balanced for age, sex, smoking history, primary disease sub-sites, HPV status, lymph node involvement, and TNM staging. The strength and the innovation of our analysis is not only shown by the rigorous matching of patient groups, and the comparison of the PT expression profile of T-with patients with the expression profile of their paired DM tissue, but also by the identification of biomarkers in primary lesions that potentially predict the development of DM. This allows spatial and temporal appreciation of HNSCC progression from the primary disease to the DM development. At first, we examined the expression levels of existing biomarkers and signatures reported as being predictive of DM. Conducting a literature survey of several independent studies, we retrieved information on 19 genes that provided evidence for their association with the spread of distant metastasis. These genes were analyzed using different methodologies at the protein (i.e., IHC, TMA) or mRNA levels (i.e., RT-qPCR). Among this list of genes, we found three of them implicated in the Epithelial Mesenchymal Transition (EMT), having an AUC > 0.6 (ANXA2, BDNF, and TGFB1). We also tested the genes included in the Rickman signature [10] that was able to stratify patients, reaching an AUC = 0.63. Taken together, our results confirm to some extent the validity of previous studies. However, they need to be improved to move towards a clinical application.

Biomarker discovery from high-dimensional genomics data involves the identification of genes that could discriminate patients belonging to clinically relevant groups. The traditional methods rely on the simple measure of the distances between data distributions while biological processes might follow complex data distributions with different shapes, intensities, and multiple modes resulting in countless ways of data crossing between densities. Therefore, we focused our attention on the modulated genes shared in T-with vs. T-without and T-with vs. DM. We applied the arrow plot procedure [14], a graphical tool based on two statistics, the overlapping coefficient (OVL) between two probability densities and the area under the receiver operating characteristic (ROC) curve (AUC), which permits to select genes either up- or down-regulated between two groups of interest, as well as “special” ones, whose average expression is similar between the two groups but may have a different distribution (i.e., multimodal).

By using the arrow plot bioinformatic approach we were able to identify new biomarkers in our cohort. By intersecting the lists of 185 and 42 differentially modulated genes obtained in the T-with vs. T-without and T-with vs. DM comparisons, respectively, we identified two genes, namely COX7A1 (which had a “*special”* trend in both comparisons) and TBX5 (which was up-regulated in both comparisons).

TBX5 and COX7A1 were tested in external independent datasets (i.e., Cromer–Rickman and TCGA) with DMFS as clinical endpoint. For outcome prediction and prognosis purposes, when a gene has a normal distribution, the median and/or some other percentile are used to cutoff the distribution and compare the groups. When we consider data having a symmetric distribution, then these are rational subgrouping ways to adopt. On the other hand, for non-symmetric or multimodal shapes, the application of those cutoffs is not an intuitive subgrouping. For this reason, we applied the Cutoff Finder tool [15], which allows optimization of a cutoff point taking into account the presence of complex distributions in the data. Our findings highlight that high expression of TBX5 and COX7A1 is associated with poor outcome in both Cromer–Rickman and TCGA datasets.

To ascertain the biological relevance of TBX5 in the metastatic process, we investigated its role in tumors other than HNSCC. DM represents the most frequent form of recurrence in the majority of breast cancer cases representing also their main cause of death. Breast cancer mainly starts as a local disease, but it can metastasize to the lymph nodes and distant organs. We tested the expression of TBX5 and its association to DMFS, providing evidence that TBX5 high expression is related to poor DMFS.

COX7A1 expression in HNSCC progression was already described by Ceder et al. [22], who explored the link of altered responsiveness of the growth-inhibitory and differentiation-inducing effects on cell-to-cell contact in tumor development. In contrast, little is known about TBX5, which is reported to be a fundamental player in the regulation of developmental processes (such as heart and limb); however, no information has been reported in the literature suggesting its involvement in HNSCC. In our dataset the expression of TBX5 increased step-by-step during the metastasis development process with low expression levels in tumors without DM, intermediate levels in tumors with DM, and high in DM tumors. Moreover, when tested in the other evaluation cohorts, its expression levels stratified patients who had a better or worst survival. A previous study in colon cancer cell lines by Yu et al. [23] suggested that TBX5 promoter hypermethylation, and the subsequent gene silencing, could be implicated in boosting proliferation and blocking apoptosis in PTs. Additionally, Zheng et al. [24] found that high levels of expression of TBX5 could predict unfavorable OS rates in gastric cancer patients with stage I and II and that TBX5 expression may be a valuable biomarker for the selection of cases of high-risk stage I and II gastric cancer. These variations in TBX5 expression levels during the progression of disease appear in line with our observations, and confirm the need of further dedicated analyses.

An examination of the changes that occur as cancer progressed revealed that there was a unique functional profile associated with each of the three statuses. In particular, the T-without tumors showed enrichment in pathways associated with the immune response, which could potentially lead to protection, supporting an avoidance of the development of DM. On the other hand, the T-with tumors, which are associated with a future metastatic event, had poor expression of immune-related pathways. When the T-with PTs were compared to their paired distant metastases, they showed increased expression in proliferation-related pathways. This could represent a first phase of the tumor invasiveness, in which the disease increases its loco-regional process of growth. Then, in comparison to PT, DMs display the classical phenotype of aggressive malignancy, delineated by high expression of pathways related to hypoxia, angiogenesis, EMT, ROS, and glycolysis [25,26,27]. As a matter of fact, in the inclusion criteria, we did not select patients with a high nodal tumor burden, but only balanced this characteristic between the two cohorts. This choice was made trying to elucidate biological pathways of distant metastatization independently from the macroscopic nodal colonization. 

As suggested by the GSEA analysis, we evaluated the involvement of the immune system comparing T-with and T-without tumors. With regards to this issue, a limitation of our analysis is that differences in circulating immune populations between T-with and T-without groups have not been evaluated. An effective systemic immune response is also crucial in preventing tumor dissemination and deserves consideration as well as the intratumor immunity [28]. Only marginal, but significant, increases in B cell plasma and T cell regulatory (Tregs) could be observed in T-with tumors. In a review by Wouters et al. [27], which mainly focused on studies in PTs, the interaction between B cells and regulatory T cells could suppress the antitumor immune responses, confirming our previous assumption. 

With regards to PD-L1, our results agreed with the gene expression profile, which showed a significantly higher level of PD-L1 expression in T-without tumor cells. This result was not confirmed by the analysis of PD-L1 in terms of CPS and IC, possibly due to the intratumor heterogeneity already described in HNSCC [29]. The highest TPS PD-L1 positivity was expected in the T-without tumors based on the available literature [30] and the highest percentage of activated inflammatory and immune-related pathways in that cohort. All of these events, acting through IFN-gamma signaling, induce tumor cells to express PD-L1 on their surface. The reason why this highest PD-L1 expression in T-without is not able to dampen all immune-activated signals is that PD-L1 is only the tip of the iceberg of the complex immune regulatory machinery [31].

The limitations of the present study are that only a relatively low number of cases were analyzed, and consequently difficulties were encountered in particular data interpretation, such as the few significantly enriched pathways in the T-with tumors, when compared to the T-without tumors, and the inconsistencies in data related to PD-L1. Thus, we think that it is important to confirm our new findings in larger number of cases, and to functionally prove that the identified genes have a role in DM development.

Despite these limitations, taking all these arguments together and by considering them in the context of previous trials with targeted agents and ongoing trials with immunotherapy as adjuvant or concurrent treatment of HNSCC, the activation of the RAS-signaling pathway has been shown to be responsible of resistance to anti-EGFR agents in recurrent and/or metastatic HNSCC [32]; nevertheless, in the present series, T-with had an intact RAS downstream pathway, therefore being theoretically more sensitive to anti-EGFR drugs. This observation is in contrast with the negative results of the trials employing adjuvant anti-EGFR tyrosine kinase inhibitors or cetuximab, that were not able to show any benefit on tumor relapses [33,34]. It is conceivable that anti-EGFR agents are not able to eliminate microresidual disease and the immune control is requested to control distant metastatization. The enrichment of immunitary pathways (“IL6 JAK STAT3 signaling”, “TNFa signaling via NFKB”, “inflammatory response”) in the T-without group is in line with this concept. We suggest integrating this gene expression analysis in the ongoing trials with immunotherapy as adjuvant treatment for HNSCC.

In summary, our results have confirmed previously published studies, and additionally reveal previously unknown events in the development of DM. If confirmed, our data may be relevant in elucidating the complex process of HNSCC metastastization as well as in identifying potentially useful molecular determinants that may allow for fine tuning of current treatments.

## 4. Materials and Methods 

### 4.1. Study Population

Clinical data, PTs, and synchronous/metachronous DMs, were retrospectively collected from 2006 to 2016 from histologically confirmed HNSCC patients at the Fondazione IRCCS Istituto Nazionale dei Tumori (INT) of Milan. Patients who developed DM are referred to as being “T-with”, whereas patients who did not develop DM are referred to as “T-without”. T-without patients were classified after a minimum follow up time of 36 months since diagnosis. T-with and T-without patients were matched according to disease sub-site, HPV status, and clinical (or pathological when available) T and N classification (according to the TNM classification used at the time of diagnosis and confirmed after revision of the VIII AJCC edition). Based on staging, early stages of disease were matched both at T and N levels (T1/T2 and N0/N1 with corresponding tumors); advanced stages were matched similarly (T3/T4 and N2/N3). HPV status was assessed only in the oropharynx sub-site, using p16 IHC and HPV DNA in situ hybridization. The study was approved by the Ethical Committee of INT of Milan (INT 57/19). The sample size of our study was calculated based on the central limit theorem: a minimum sample size of 25 to 30 per classes is necessary for data distribution to be close to a normal distribution.

### 4.2. Whole Transcriptome Analysis

Gene expression analysis was performed on FFPE tumor tissue samples. RNA was extracted using the miRNeasy FFPE kit (Qiagen, Hilden, Germany), according to the manufacturer’s instructions. Quantification of extracted RNA was performed using Qubit 2.0 Fluorimetric Assay (Thermo Fisher Scientific, Waltham, MA, USA), and the quality of nucleic acid was assessed using a TapeStation4200 (Agilent Technologies, Santa Clara, CA, USA). Gene expression experiments were performed using the Whole-Genome DASL (cDNA-mediated Annealing, Selection, Extension, and Ligation) assay and HumanHT12_v4 BeadChips (Illumina, San Diego, CA, USA) which covered more than 29,000 annotated genes derived from RefSeq (Build 36.2, Release 38). The chips were scanned and primary data were recovered using BeadScan (Illumina). Data were processed and quantile-normalized using BeadStudio software (Illumina); to filter the reliable data, a detection *p*-value < 0.05 was set for each gene, yielding an expression matrix containing 15,868 probe-sets. 

### 4.3. Biomarker Testing and Identification

In order to test the signature developed by Rickman et al. [10] we retrieved the list of 19 genes that was used to construct their model. Seventeen out of 19 of these genes were present in our microarray profiling and so were used to test the signature. These genes were: GPRASP2, FLOT2, YIPF6, GUK1, KRT17, LAMB3, ATP2B4, HSD17B12, C6orf107, GLT28D1, PSMD10, ZNF77, DHX35, ARMCX5, SYBL1, BHLHB9, and KIAA1729. In order to assess the variations in the signature over individual samples, a Gene Set Variation Analysis (GSVA) was applied by imputing gene set enrichment scores for each sample using the Pathway Level Analysis of Gene Expression (PLAGE) as the enrichment method [35]. Through a survey of the literature we also collected data for 19 genes whose expression has been previously found to be associated with the development of metastasis: 16 of these genes were studied by IHC (AKT1, ANXA2, BDNF, BIRC5, BMI1, CDH1, CRYAB, DSP, FER1L3, IL1A, NBN, NOTCH1, NTRK2, S100A4, TGFB1, and TWIST1), and the remaining 3 at the mRNA level (MPO, NTS, and NTSR1). These genes were tested for their ability to discriminate between T-with and T-without cases.

The arrow plot [14] was used to identify potential biomarker genes showing accuracy in defining the groups of interest (i.e., T-with vs. T-without or T-with vs. DM). The method is an exploratory analysis tool, which combines two non-parametric measures visualized in a two-dimensional plot of the overlapping coefficient (OVL) as the x-coordinate and the area under the curve (AUC) value as the y-coordinate. The area under the ROC curve (AUC) is the most frequently used ROC (receiver operating characteristic) index. An ROC curve is used to establish the discriminatory accuracy of a biomarker in distinguishing between two conditions [36]: AUC values close to 1 (or to 0) represent a ‘good’ classifier with up-regulation (or down-regulation). However, ROC curves fail to identify biomarkers whose expression has a different data distribution but AUC close to 0.5 [37]. The overlapping coefficient (OVL) can solve this issue by assessing the common area between probability density functions. In this way, OVL explains whether a gene with an AUC close to 0.5 can be of interest (low value for the overlapping coefficient) or not (high value for the overlapping coefficient). By plotting the OVL and AUC values for every gene on the x- and y-axes, respectively, we were able to construct the arrow plot. By choosing different threshold levels for the OVL and AUC, we could select genes differentiating the two groups as well as the “special genes” to be potential biomarkers. Special genes have peculiar data distribution between the groups under investigation characterized by similar means but different variances. OVL and AUC values for the arrow plot were computed with the overlapping [38] and ROC R packages, respectively. As a threshold to filter genes, we imposed an OVL < 0.4 and AUCs of > 0.75 or < 0.25 for the up- and down-regulated genes, respectively, while the special genes were selected by the area defined by an OVL < 0.4 and 0.4 < AUC < 0.6.

### 4.4. Evaluation of the Newly Identified Biomarkers on External Datasets

To test TBX5 and COX7A1, two public datasets reporting DMFS that have been profiled on different versions of Affymetrix array chips were selected. These were from Rickman et al. [10] and available at ArrayExpress (https://www.ebi.ac.uk/arrayexpress/; ID: E-TABM-302) including 81 cases, and from Cromer et al. [7] available at GEO (https://www.ncbi.nlm.nih.gov/geo/; ID: GSE2379) including 31 cases. The two datasets were integrated using a meta-analysis approach. Signal intensities were normalized within each individual dataset using the Robust Multi-Array Average (RMA) method and after annotation, redundant probe sets were collapsed using EntrezID and the “maxRowVariance” method (function: collapseRows) of the WGCNA package version 1.63 [39]. Batch effects were removed using ComBat [40] to generate a final data matrix containing 112 cases and 8400 unique genes.

To further evaluate TBX5 and COX7A1, we took into consideration TCGA data. A recent work re-annotates the TCGA clinical-pathologic data developing a standardized database named the TCGA Pan-Cancer Clinical Data Resource [41]. After retrieving the annotated clinical data, TCGA HNSCC reported 48 patients having DM and 80 who developed loco-regional recurrence. The “RSEM_genes_normalized” RNAseq TCGA data were retrieved from FireBrowse (BROAD institute; https://gdac.broadinstitute.org/).

For biomarker cutoff determination, we used the Cutoff Finder R package available at http://molpath.charite.de/cutoff [15] that allows to optimize a cutoff point taking into account the presence of complex distributions in the data. We applied Cutoff Finder to optimize the correlation with DMFS data; in this case, the survival analysis was performed using the coxph and survfit functions of the survival R package [42]. A Cox proportional hazard model was fitted to the dichotomized variable and the survival data. The point with the most significant (log-rank test) split was considered as the optimal cutoff. Hazard ratios (HRs) including 95% confidence intervals were calculated and plotted in the range of biomarker expression. To test the role of a selected gene in predicting DM in other diseases, the KM Plotter Tool (http://kmplot.com/analysis/) [16,17] was used to calculate hazard ratios, and log-rank p-values for the aggregated breast cancer clinical studies. The “auto select best cutoff” function of KM Plotter was used to stratify the cases. Representative Affymetrix probes were selected by Jetset [43] corresponding to 240715_at for TBX5 in 664 breast cancer cases and DMFS was used as clinical endpoint.

### 4.5. Functional Bioinformatic Analyses

Bioinformatic analyses included: i) a gene set enrichment analysis (GSEA) [18,19] aimed at recognizing the enriched gene sets from the Hallmark collection belonging to the Molecular Signatures Database (MSigDB) that summarized well-known biological processes; ii) an assessment of tumor microenvironment determinants, using the CIBERSORT procedure in absolute mode [20] which estimates the proportion of 22 immune cell populations. 

We performed a pre-ranked version of GSEA by running GSEA v3.0 [build: 0160] with default parameters. The ranked list was obtained by comparing groups of samples with the R package limma [44] and then sorting the genes according to the value of their moderated t-statistic. The CIBERSORT procedure was performed using the immunedenconv R package [45].

For statistical analyses, we used R software version 3.6.0 and Bioconductor version 3.9 [46,47]. Plots were produced using the R package ggplot2 [48] along with its extension ggrepel (https://ggrepel.slowkow.com/), ComplexHeatmap [49], and ggradar (https://github.com/ricardo-bion/ggradar), and were successively assembled into panels using the free and open source vector graphics editor InkScape [50]. We performed a Wilcoxon rank sum tests for every immune cell population with a level of significance of α = 0.05. 

### 4.6. PD-L1 detection and TILs Evaluation

The PD-L1 evaluation was performed on 20 T-with and 21 T-without cases; 5 cases in T-with group and 3 in the T-without group were excluded from the evaluation due to tissue exhaustion. PD-L1 IHC using the PD-L1 IHC 22C3 pharmDx kit (Dako, Carpinteria, CA, USA) on the Dako ASL48 platform was performed on FFPE tumor sections, according to the manufacturer’s recommendations. PD-L1 expression was evaluated both in tumor and inflammatory cells. Briefly, the percentage of tumor cells showing PD-L1 membrane immunoreactivity at any intensity determined the tumor proportion score (TPS). Staining in tumor-associated inflammatory cells (IC) (including macrophages, dendritic cells, plasma cells, histiocytes, and lymphocytes) was scored as the percentage area of PD-L1–stained ICs present in the area occupied by any IC.

Following this, a combined proportion score (CPS) was determined, which was defined as the number of PD-L1 staining cells (tumor cells, lymphocytes, macrophages) divided by the total number of viable tumor cells, multiplied by 100. A minimum of 100 viable tumor cells had to be present in the PD-L1 stained slide to be considered adequate for PD-L1 evaluation. For the present analysis, we considered samples showing PD-L1 immunoreactivity in more than 1% of tumor cells (TPS) or immune cells (IC score) as being positive. A CPS score equal to or greater than 1 was considered positive.

Tumor infiltrating lymphocytes (TILs) were assessed on hematoxylin and eosin (H and E) slides, as previously described [51,52]. Briefly, mononuclear inflammatory cells (i.e., lymphocytes and plasma cells) present in the stromal compartment were assessed. The percentage of stromal TILs was calculated as the area of stromal tissue (within and at the invasive edge of tumor area) occupied by inflammatory cells over the total.

### 4.7. Data Availability

Microarray data were compliant to MIAME (Minimum Information about a Microarray Experiment) and the gene expression profiles of our cohort (*n* = 72; 24 T-without, 25 T-with, and 23 DM) were deposited on GEO (accession number GSE136037).

## 5. Conclusions

With the final goals of identifying biomarkers that could be used to predict patients who will develop DM and to explore the biology behind metastasis, we initially tested available signatures/genes. Since the performance of these potential biomarkers did not reach a level to enter into the clinical practice, we decided to explore a new analytical approach (i.e., arrow plot). We identified TBX5 and COX7A1 as associated with DM; our findings were then tested on three independent datasets and high expression of both genes was associated with worst outcome.

Biological functional analyses pointed out that the immune control is requested to control DM. The enrichment of immune pathways (“IL6 JAK STAT3 signaling”, “TNFa signaling via NFKB”, “inflammatory response”) in the T-without group highlighted this concept, suggesting a future integration of these genes in the ongoing trials with immunotherapy as adjuvant treatments for HNSCC.

If confirmed, our results may be useful in better understanding the complex process of HNSCC metastasis as well as in identifying novel molecular determinants that may allow for a fine improvement of current treatments.

## Figures and Tables

**Table 1:** Demographic and clinical characteristics. *p*-values were computed by Fisher’s exact tests (categorical data) and Wilcoxon rank-sum tests (numerical data).

Demographic and Clinical Characteristics			
**At diagnosis**			
**Sex**			0.289
M	22	18	
F	3	6	
**Median age (years)**	60 (range: 43–76)	63 (range: 43–77)	0.1124
**Smoking history**			0.7163
Yes	17	19	
No	6	3	
NA	2	2	
**Sub-site**			0.9543
Oropharynx	7	8	
Larynx	8	8	
Oral Cavity	7	5	
Hypopharynx	3	3	
**HPV status**			0.2
Positive	6	5	
Negative	-	3	
Unknown	1	-	
**TNM (VIII ed.)**			0.7416
I-II	7	5	
III-IV	18	19	
**Local Lymph Node Involvement**			0.7733
Yes	16	14	
No	9	10	
**At Follow-Up**			
	**T-with (*n* = 25)**	**T-without (*n* = 24)**	***p*** **-value**
**DM sub-site**			
Lung	13	-	
Liver	3	-	
Other	9	-	
**Loco-regional recurrence**			
Yes	12	-	
No	13	-	
**Median time (months) to**	**DM development**	**Follow-up**	3.19 × 10^−9^
	16 (range: 3–59)	67 (range: 48–106)	

## Supplementary Materials

The following are available online at https://www.mdpi.com/2072-6694/12/4/922/s1, Figure S1: Lymph-node involvement at the time of diagnosis; Figure S2: Receiver operating characteristic (ROC) curve for the Rickman signature designed to predict distant metastasis; Figure S3: TBX5 and COX7A1 in HNSCC TCGA dataset. Figure S4: TBX5 in breast cancer; Table S1: Biomarkers for the prediction of distant metastasis in primary tumors; Table S2: List of genes selected by AUC and OVL in comparison of the T-with vs. T-without groups and T-with vs. DM groups; Table S3: Main clinical features of the combined Cromer–Rickman dataset; Table S4: List of gene-sets enriched in the T-with vs. T-without comparison; Table S5: List of gene-sets enriched in the T-with vs. DM comparison; Table S6: PD-L1 detection and Immune landscape: PD-L1 detection and evaluation of TILs.
