# Supplementary material for: Tumor Biomarkers for the Prediction of Distant Metastasis in Head and Neck Squamous Cell Carcinoma
Source: Cancers (Basel). 2020 Apr 9;12(4):922. doi: 10.3390/cancers12040922 (PMC7225924; doi:10.3390/cancers12040922)
Supplement: Supplementary file 1 [file cancers-12-00922-s001.zip › cancers-766464-supplementary-final 2/Supplementary-Figures and tables_rev2.docx]

Tumor Biomarkers for the Prediction of Distant Metastasis in Head and Neck Squamous Cell Carcinoma

Salvatore Alfieri, Andrea Carenzo, Francesca Platini, Mara S. Serafini, Federica Perrone, Donata Galbiati, Andrea P. Sponghini, Roberta Depenni, Andrea Vingiani, Pasquale Quattrone, Edoardo Marchesi, Maria F. Iannó, Arianna Micali, Elisa Mancinelli, Ester Orlandi, Sara Marceglia, Laura D. Locati, Lisa Licitra, Paolo Bossi and Loris De Cecco


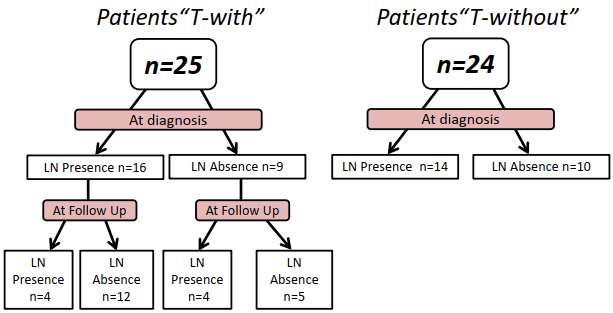


**Figure S1.** Lymph-node involvement at the time of diagnosis.


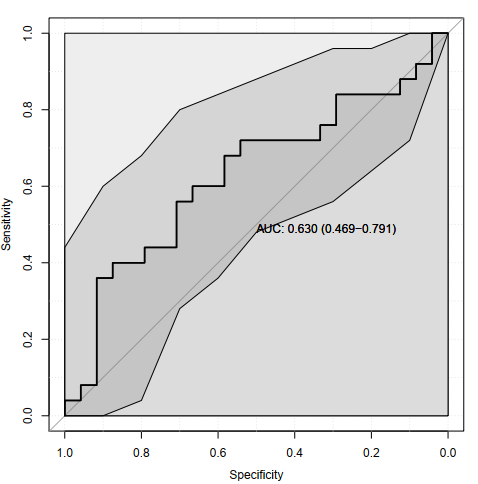


**Figure S2.** Receiver operating characteristic (ROC) curve for the Rickman signature designed to predict distant metastasis.


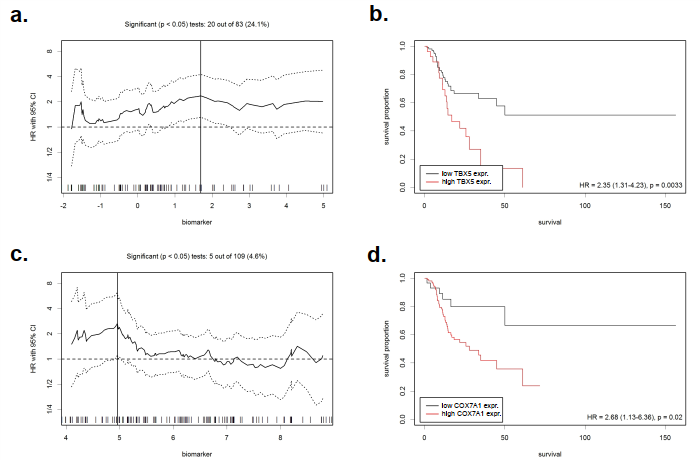


**Figure S3.** TBX5 and COX7A1 in HNSCC TCGA dataset.


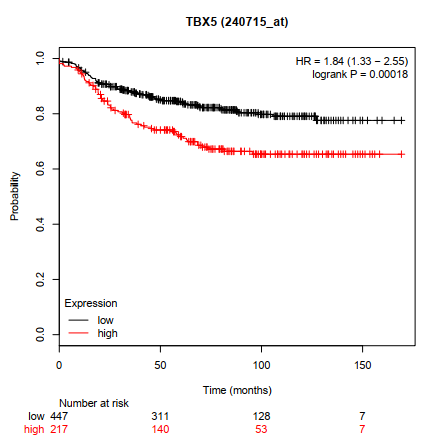


**Figure S4.** TBX5 in breast cancer.

**Table S1.** Biomarkers for the prediction of distant metastasis in primary tumors.

| **Entrez ID** | **Gene Symbol** | **OVL_T-with vs T-without** | **AUC_T-with vs T-without** | **Assay** | **Subsite** | **Reference** |
| --- | --- | --- | --- | --- | --- | --- |
| 26509 | FER1L3 | 0.710 | 0.528 | IHC | OPSCC | [1] |
| 7291 | TWIST1 | 0.643 | 0.420 | IHC | all subsites | [2] |
| 207 | AKT1 | 0.666 | 0.525 | IHC | all subsites | [3] |
| 332 | BIRC5 | 0.755 | 0.483 | IHC | all subsites | [3] |
| 648 | BMI1 | 0.602 | 0.347 | IHC | LSCC | [4] |
| 1410 | CRYAB | 0.883 | 0.455 | IHC | all subsites | [5] |
| 1832 | DSP | 0.791 | 0.486 | IHC | OSCC | [6] |
| 3552 | IL1A | 0.640 | 0.420 | IHC | all subsites | [7] |
| 999 | CDH1 | 0.664 | 0.528 | IHC | laryngeal and hypopharyngeal | [8] |
| 302 | ANXA2 | 0.570 | 0.605 | IHC | laryngeal and hypopharyngeal | [8] |
| 4353 | MPO | 0.425 | 0.532 | mRNA | all subsites | [9] |
| 6275 | S100A4 | 0.765 | 0.467 | IHC | OSCC | [10] |
| 4922 | NTS | 0.656 | 0.538 | mRNA | all subsites | [11] |
| 4923 | NTSR1 | 0.799 | 0.479 | mRNA | all subsites | [11] |
| 4915 | NTRK2 | 0.840 | 0.485 | IHC | all subsites | [12] |
| 627 | BDNF | 0.567 | 0.722 | IHC | all subsites | [12,13] |
| 7040 | TGFB1 | 0.561 | 0.717 | IHC | all subsites | [12,14] |
| 4851 | NOTCH1 | 0.691 | 0.587 | IHC | hypopharyngeal | [15] |
| 4683 | NBN | 0.681 | 0.465 | IHC | all subsites | [16] |

**Table S3.** Main clinical features of the combined Cromer–Rickman dataset.

|  | **Validation (N = 112)** |
| --- | --- |
| **Sex** |  |
| M | 105 |
| F | 7 |
| **Median age (at diagnosis)** | 59 (35-79 years) |
| **Subsite** |  |
| Oropharynx | 17 |
| Larynx | 0 |
| Oral Cavity | 28 |
| Hypopharynx | 67 |
| **Post surgical treatment** |  |
| RX | 99 |
| RX+CT | 12 |
| N | 1 |
| **Differentiation** |  |
| 1 | 26 |
| 2 | 56 |
| 3 | 30 |

**Table S4. List of gene-sets enriched in the T-with vs. T-without comparison.** Differentially enriched pathways in T-with compared to T-without groups: GSEA Hallmark analysis.

|  | **HALLMARK**  **Geneset name** | **Total**  **genes^a^** | **NES^b^** | **Nom**  **p-value** | **FDR**  **q-val** |
| --- | --- | --- | --- | --- | --- |
| **Enriched in T-without** | | | | | |
|  | IL6 JAK STAT3 SIGNALING | 79 | -1.89 | 0.003 | 0.011 |
|  | PROTEIN SECRETION | 92 | -1.86 | < 0.001 | 0.006 |
|  | TNFA SIGNALING VIA NFKB | 187 | -1.77 | < 0.001 | 0.009 |
|  | ANDROGEN RESPONSE | 94 | -1.72 | < 0.001 | 0.015 |
|  | MYC TARGETS V1 | 191 | -1.71 | < 0.001 | 0.012 |
|  | KRAS SIGNALING UP | 178 | -1.68 | < 0.001 | 0.012 |
|  | INFLAMMATORY RESPONSE | 182 | -1.56 | < 0.001 | 0.031 |
|  | UV RESPONSE DN | 133 | -1.48 | < 0.001 | 0.055 |
| **Enriched in T-with** | | | | | |
|  | PANCREAS BETA CELLS | 27 | 1.45 | 0.060 | 0.076 |
|  |  |  |  |  |  |

GS: geneset; thresholds: FDR < 0.1; |NES|≥1.4; Number of total genes present in the geneset, ^b^ NES = normalized enrichment score; T without= primary tum che non ha sviluppato (non-metastasized HNSCC); T with= primary tum che ha sviluppato distant metastasized HNSCC.

**Table S5. List of gene-sets enriched in the T-with vs. DM comparison.** Differentially enriched pathways in primary tumors compared to paired distant metastasis groups: GSEA Hallmark analysis.

|  | **HALLMARK**  **Geneset name** | **Total**  **genes^a^** | **NES^b^** | **Nom**  **p-value** | **FDR**  **q-val** |
| --- | --- | --- | --- | --- | --- |
| **Enriched in Primary** | | | | | |
|  | E2F TARGETS | 184 | -1.89 | 0.003 | 0.011 |
|  | G2M CHECKPOINT | 183 | -1.86 | < 0.001 | 0.006 |
|  | ALLOGRAFT REJECTION | 180 | -1.77 | < 0.001 | 0.009 |
| **Enriched in Metastasis** | | | | | |
|  | HYPOXIA | 187 | 2.12 | < 0.001 | 0.001 |
|  | ANGIOGENESIS | 32 | 1.86 | < 0.001 | 0.003 |
|  | EPITHELIAL MESENCHYMAL TRANSITION | 193 | 1.85 | < 0.001 | 0.009 |
|  | XENOBIOTIC METABOLISM | 175 | 1.82 | < 0.001 | 0.002 |
|  | REACTIVE OGEN SPECIES PATHWAY | 47 | 1.80 | < 0.001 | 0.003 |
|  | COAGULATION | 114 | 1.66 | 0.002 | 0.009 |
|  | GLYCOLYSIS | 187 | 1.64 | < 0.001 | 0.011 |
|  | HEME METABOLISM | 172 | 1.62 | < 0.001 | 0.013 |
|  | HEDGEHOG SIGNALING | 32 | 1.51 | 0.038 | 0.035 |
|  | ESTROGEN RESPONSE EARLY | 175 | 1.49 | 0.002 | 0.039 |
|  | UV RESPONSE UP | 147 | 1.48 | 0.002 | 0.037 |
|  | APICAL JUNCTION | 181 | 1.42 | 0.015 | 0.065 |
|  | ADIPOGENESIS | 190 | 1.41 | 0.007 | 0.062 |
|  |  |  |  |  |  |

GS: geneset; thresholds: FDR < 0.1; |NES|≥1.4; ^a^ Number of total genes present in the geneset; ^b^ NES = normalized enrichment score.

Reference

1. Kumar, B.; Brown, N.V.; Swanson, B.J.; et al. High expression of myoferlin is associated with poor outcome in oropharyngeal squamous cell carcinoma patients and is inversely associated with HPV-status. *Oncotarget* **2016**, *7*, 18665–18677.
2. Zhuo, X.; Luo, H.; Chang, A.; Li, D.; Zhao, H.; Zhou, Q. Is overexpression of TWIST, a transcriptional factor, a prognostic biomarker ofhead and neck carcinoma? Evidence from fifteen studies. *Sci. Rep.* **2015**, *5*, 18073.
3. Pickhard, A.; Grober, S.; Haug, A.K.; et al. Survivin and pAkt as potential prognostic markers in squamous cell carcinoma of the head and neck. *Oral Surg. Oral Med. Oral Pathol. Oral Radiol.* **2014**, *117*, 733–742.
4. Allegra, E.; Trapasso, S.; Pisani, D.; et al. The role of BMI1 as a biomarker of cancer stem cells in head and neck cancer: a review. *Oncology* **2014**, *86*, 199–205.
5. Van de Schootbrugge, C.; Bussink, J.; Span, P.N.; Sweep, F.C.; Grénman, R.; Stegeman, H.; Pruijn, G.J.; Kaanders, J.H.; Boelens, W.C. αB-crystallin stimulates VEGF secretion and tumor cell migration and correlates with enhanced distant metastasis in head and neck squamous cell carcinoma. *BMC Cancer* **2013**, *13*, 128.
6. Papagerakis, S.; Shabana, A.H.; Pollock, B.H.; Papagerakis, P.; Depondt, J.; Berdal, A. Altered desmoplakin expression at transcriptional and protein levels provides prognostic information in human oropharyngeal cancer. *Hum. Pathol.* **2009**, *40*(9), 1320–1329. doi: 10.1016/j.humpath.2009.02.002.
7. León, X.; Bothe, C.; García, J.; Parreño, M.; Alcolea, S.; Quer, M.; Vila, L.; Camacho, M. Expression of IL-1α correlates with distant metastasis in patients with head and neck squamous cell carcinoma. *Oncotarget* **2015**, *10*, 6(35), 37398–37409. doi: 10.18632/oncotarget.6054.
8. Rodrigo, J.P.; Martínez, P.; Allonca, E.; Alonso-Durán, L.; Suárez, C.; Astudillo, A.; García-Pedrero, J.M. Immunohistochemical markers of distant metastasis in laryngeal and hypopharyngeal squamous cell carcinomas. *Clin. Exp. Metastasis* **2014**, *31*, 317–325.
9. Lopez-Pousa, A.; Sumarroca, A.; Quer, M.; Camacho, M.; Garcia, J.; Lopez, M.; Duenas Cid, N.; Pavon, M.A.; Farre, N.; Gallego Rubio, O.; et al. Risk of distant metastases in head and neck carcinoma patients and myeloperoxidase (MPO) expression*. J.Clin. Oncol.* **2016**, *34*, 6067–6067
10. Natarajan, J.; Hunter, K.; Mutalik, V.S.; Radhakrishnan, R. Overexpression of S100A4 as a biomarker of metastasis and recurrence in oral squamous cell carcinoma. *J. Appl. Oral Sci.* **2014**, *22*(5), pp.426–433.
11. Shimizu, S.; Tsukada, J.; Sugimoto, T.; Kikkawa, N.; Sasaki, K.; Chazono, H.; Hanazawa, T.; Okamoto, Y.; Seki, N. Identification of a novel therapeutic target for head and neck squamous cell carcinomas: a role for the neurotensin-neurotensin receptor 1 oncogenic signaling pathway. *Int. J. Cancer* **2008**, *123*(8), 1816–1823. doi: 10.1002/ijc.23710.
12. Leemans, C.R.; Braakhuis, B.J.; Brakenhoff, R.H. The molecular biology of head and neck cancer*. Nat. Rev. Cancer* **2011**, *11*(1), 9–22. doi: 10.1038/nrc2982.
13. Kupferman, M., Jiffar, T., El-Naggar, A. et al. TrkB induces EMT and has a key role in invasion of head and neck squamous cell carcinoma. *Oncogene* **2010**, *29*, 2047–2059, doi:10.1038/onc.2009.486
14. Cohen, J. et al. Attenuated transforming growth factor β signaling promotes nuclear factor-κB activation in head and neck cancer. *Cancer Res.* **2009**, *69*, 3415–3424.
15. Tian, J.; Liu, X.; Liu, X.; Jing, P.; Sa, N.; Wang, H.; Xu, W. Notch1 serves as a prognostic factor and regulates metastasis via regulating EGFR expression in hypopharyngeal squamous cell carcinoma. *Onco. Targets Ther.* **2018**, *11*, 7395–7405. doi: 10.2147/OTT.S175423.
16. Yang, M.H.; Chang, S.Y.; Chiou, S.H.; Liu, C.J.; Chi, C.W.; Chen, P.M.; Teng, S.C.; Wu, K.J. Overexpression of NBS1 induces epithelial–mesenchymal transition and co-expression of NBS1 and Snail predicts metastasis of head and neck cancer. *Oncogene* **2007**, *26*(10), pp.1459–1467.
